# The emerging roles of WBP2 oncogene in human cancers

**DOI:** 10.1038/s41388-020-1318-0

**Published:** 2020-05-11

**Authors:** Hossein Tabatabaeian, Angad Rao, Alisha Ramos, Tinghine Chu, Marius Sudol, Yoon Pin Lim

**Affiliations:** 1 Cancer Science Institute of Singapore, National University of Singapore, Singapore, 117599 Singapore; 2 Department of Biochemistry, Yong Loo Lin School of Medicine, National University of Singapore, Singapore, 117545 Singapore; 3 NUS Graduate School for Integrative Sciences and Engineering, National University of Singapore, Singapore, 117456 Singapore; 4 Department of Physiology, National University of Singapore, Mechanobiology Institute, Singapore, 117597 Singapore; 5 Institute for Molecular and Cell Biology (IMCB, A*STAR), Singapore, 138673 Singapore; 6 Icahn School of Medicine at Mount Sinai, New York, NY 10029 USA; 7 National University Cancer Institute, Singapore, 119082 Singapore

**Keywords:** Oncogenes, Growth factor signalling

## Abstract

WW domain-binding protein 2 (WBP2) is an emerging oncoprotein. Over the past decade, WBP2 surfaced as a key node connecting key signaling pathways associated with ER/PR, EGFR, PI_3_K, Hippo, and Wnt in cancer. In addition to the oncogenic functions of WBP2, this review discusses the latest research regarding the multilevel regulation and modes of action of WBP2 and how they can be exploited for molecular medicine. In translational research, evidence supports the role of WBP2 as a biomarker for early detection, prognosis, and companion diagnostics in breast cancer. Finally, we envision new trends in WBP2 research in the space of molecular etiology of cancer, targeted therapeutics, and precision medicine.

## Historical background of WW domain-binding protein 2 (WBP2)

Cancer is expected to rank as the number one cause of death globally in the 21st century overtaking cardiovascular diseases [1]. While the past decades have seen a wealth of knowledge added to our understanding and clinical management of cancer, there is also increasing awareness of the need for higher resolution, more precise molecular and medical research in the areas of early detection, targeted therapeutics, and personalized medicine. Discovery of novel oncogenes and delineation of the function, regulation, and mechanism of these oncogenes has the potential to usher new and better biomarkers as well as better strategies in rational drug development.

**Fig. 1:**
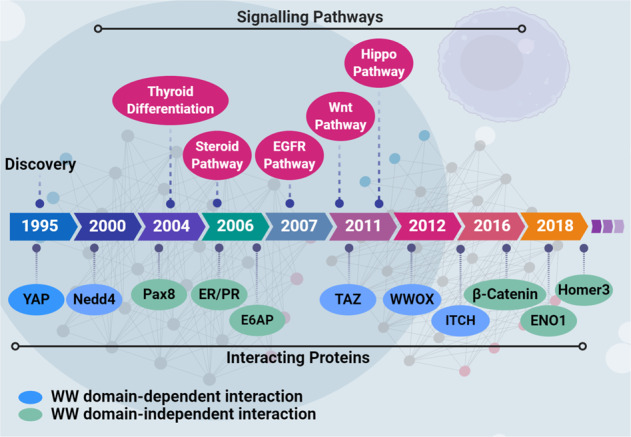
Schematic timeline of WBP2 historical background. WBP2’s interactions with Yes-associated Protein (YAP) [2], Neural precursor cell expressed, developmentally downregulated 4 E3 ubiquitin-protein ligase (Nedd4) [64], paired box 8 (Pax8) [94], progesterone receptor (PR) [5], TAZ [12], E6-associated protein (E6AP) [5], WW domain-containing oxidoreductase (WWOX) [65], estrogen receptor (ER) [4], β-catenin [13], and itchy E3 ubiquitin-protein ligase (ITCH) [13] identified from 1995 to 2016, with the corresponding signaling pathways including Hippo [12], steroid receptor [4, 5], EGFR [10], and Wnt [11].

WBP2 was initially identified as a partner of Yes-associated Protein (YAP) in 1995 [2]. Although more than 20 years have passed, the momentum in WBP2 research has only begun to accelerate in recent years. This could be due to the fact that there was limited information on the role of WBP2 in human diseases. Ten years since 1995, the progress in WBP2 research was mainly in the biochemistry space while the next 10 years were characterized by the implication of WBP2 in signal transduction pathways. A chronological account of the findings associated with WBP2 research depicting its interacting proteins and the cellular signaling pathways WBP2 is involved in is shown in Fig. 1.

The cellular and molecular role of WBP2 protein, especially in the context of the steroid pathway, has been recently reviewed by Chen et al. [3]. This review focuses on the more recent advances in the oncogenic function and regulation of WBP2 oncoprotein, as well as its potential utilities in cancer detection, therapy, and precision medicine.

## WBP2 function

Following biochemical characterization of WBP2 resulting in the knowledge of its binding partners and involvement in steroid signaling [4, 5], WBP2 has since been discovered to play roles in a variety of human diseases such as hearing loss and infertility [6, 7]. However, it is in the area of cancer where the investigations have been most intense. Together with YAP and TAZ, WBP2 belongs to a growing family of oncogenic transcription coactivators [8, 9].

### WBP2 in human cancer

#### In vitro evidence for breast cancer

WBP2 was first discovered by our group to be associated with breast cancer in 2007 when it was shown by phosphoproteomic profiling to be hyperphosphorylated in an isogenic MCF-10AT breast cancer progression model comprising 4 isogenic xenograft-derived human cell lines that mimic different stages of breast cancer progression [10]. Subsequently, we and others demonstrated WBP2 to possess oncogenic/tumorigenic properties such as the promotion of anchorage-independent growth and invasiveness to breast epithelial cells [11–13]. Our follow-up study revealed that the expression of WBP2 is higher in Epidermal Growth Factor Receptor 2 (HER2)^+^ and triple-negative breast cancer (TNBC) cell lines compared with the less aggressive estrogen receptor (ER)^+^ cell lines like MCF-7 [13]. Consistently, Song et al. demonstrated an oncogenic function of WBP2 in promoting survival and growth of TNBC cell lines [14].

#### Ex vivo/clinical evidence for breast cancer

The clinical significance of WBP2 was demonstrated when we analyzed >400 breast tissue samples and showed that WBP2 has higher expression in >85% of the tumors as compared with normal tissues [13]. Specifically, the 439 samples comprised a spectrum of lesions including non-cancer, benign, hyperplasia, ductal carcinoma in situ (DCIS), invasive ductal carcinoma, and metastatic tissues. Nuclear WBP2, which is the predominant oncogenic form as will be discussed more later, was scored following immunohistochemistry (IHC). Remarkably, three quarter or more of the invasive and metastatic cases showed moderate to high IHC score. On the other hand, most of the non-cancer tissues showed no nuclear WBP2 while the majority of the preneoplastic DCIS samples displayed low to moderate nuclear WBP2 expression. That aberrant WBP2 expression could be detected in DCIS, a preneoplastic lesion, and its elevated levels in invasive/metastatic cancer suggest that WBP2 plays a role in disease initiation and progression. Nuclear WBP2 level was also tested for its correlation with tumor grade and size. Grade 2 and 3 tumors were found to have statistically higher nuclear WBP2 levels compared with grade 1 tumor. Similarly, small (0–2 cm), moderate (2–5 cm), and large (>5 cm) tumors showed increasing levels of nuclear WBP2. Finally, nuclear WBP2 levels correlated inversely with disease-free and overall survival of breast cancer patients [13]. Cytoplasmic level of WBP2 also correlated with the above histological-clinical parameters, albeit less strongly. These observations position WBP2 as a potential biomarker for early detection and prognosis of cancer.

Our latest study on WBP2 and HER2 expression in 296 resected breast tumor tissues revealed that the levels of WBP2 and HER2 were positively correlated [15]. HER2^+^ patients whose tumors showed high nuclear WBP2 expression (as determined by IHC) had the worst overall and disease-free survival than other groups. The data supports the notion that WBP2 expression in combination with HER2 is more powerful than either alone for breast cancer prognosis.

#### WBP2 in other human cancers

WBP2’s role in other human cancer types has also been reported. High expression of WBP2 in cutaneous squamous cell carcinoma is associated with higher proliferation and clonal growth. WBP2 was shown as a key driver of proliferation in human epithelial stem cells [16]. Elevated WBP2 expression has also been reported in human gliomas and exogenous WBP2 increased cell proliferation, migration, and cell cycle progression [17]. More recently, gain/loss-of-function studies demonstrated an oncogenic role of WBP2 in hepatocellular carcinoma [18]. Collectively, WBP2 possesses oncogenic properties in an increasing number of human cancer types as summarized in Table 1.

**Table 1:** Implication of WBP2 protein in different cancers.

Cancer	Method/approach	Findings	Year	Refs.*
Breast	• Phosphoproteomics of MCF-10AT isogenic breast cancer progression model.	• WBP2 phosphoprotein level increased during breast cancer progression.	2007	[10]
	• In vitro cell-based assays.	• WBP2 cooperated with TAZ to promote cellular transformation in MCF-10A.	2011	[12]
	• In vitro cell-based assays. • Xenograft model.	• WBP2 overexpression transformed non-cancer cells, increased EMT, migration, and invasion of low-grade MCF-7 cancer cells. • WBP2 overexpression promoted tumorigenesis in nude mice.	2011	[11]
	• In vitro cell-based assays. • Xenograft model. • Clinical sample studies.	• Higher WBP2 expression in a panel of breast cancer cell lines including HER2^+^ and TNBC compared with normal mammary epithelial cells. • WBP2 expression is elevated in clinical breast cancer and correlated with tumor grade and size. • WBP2 knockdown reduced tumor volume in mice, and decreased 3D cell growth and invasion.	2016	[13]
	• In vitro cell-based assays.	• WBP2 promoted cell cycle progression in low-grade MCF-7 cancer cells.	2017	[19]
	• In vitro cell-based assays. • Xenograft model.	• Overexpression of WBP2 increased chemoresistance to doxorubicin in low-grade MCF-7 breast cancer cell lines, and xenograft mice.	2018	[20]
	• In vitro cell-based assays. • Clinical sample analysis.	• WBP2 is overexpressed in TNBC samples. • WBP2 downregulation inhibited TNBC proliferation by blocking YAP transcription and EGFR/PI_3_K/Akt signaling pathway	2018	[14]
	• In vitro cell-based assays. • Clinical sample analysis.	• WBP2 cooperated with USF-1 to promote proliferation of low-grade MCF-7 cells.	2018	[21]
	• In vitro cell-based assays.	• WBP2 promoted TNBC via the JNK/Jun kinase pathway	2018	[22]
	• Clinical sample analysis. • Xenograft model.	• Patients with WBP2^+^/ HER2^+^ breast cancer had worse prognosis than either alone. • WBP2 overexpression sensitized breast tumor to trastuzumab in mice.	2019	[15]
cSCC	• In vitro cell-based assays.	• WBP2 identified as an important co-factor of YAP-mediated gene transcription via RNAi screen. • WPB2 expression was upregulated in actively proliferating cSCC cells and downregulated during terminal differentiation. • WBP2 knockdown reduced the clonal growth of SCC13 cells.	2017	[23]
Glioma	• In vitro cell-based assays. • Xenograft model.	• WBP2 overexpression promoted glioma via the ENO1/PI_3_K/Akt pathway.	2018	[24]
Canine oral	• Mass spectrometry.	• WBP2 shown to be overexpressed in all types of canine oral tumors.	2018	[25]
HCC	• In vitro cell-based assays. • Xenograft model. • Clinical sample analysis.	• WBP2 shown to be highly expressed in hepatocellular carcinoma clinical samples. • MicroRNA-485-5p inhibited hepatocellular carcinoma by blocking the WBP2/Wnt signaling pathway.	2020	[18]

* Papers are included if they support WBP2’s involvement in cancer based on the following evidence i) changes in WBP2 expression between normal and disease states or ii) a functional phenotype in in vitro or in vivo assays. *cSCC* cutaneous squamous cell carcinoma, *HCC* hepatocellular carcinoma, *TNBC* triple-negative breast cancer, *WBP2* WW domain-binding protein 2.

### Multimodal action of WBP2 protein

**Fig. 2:**
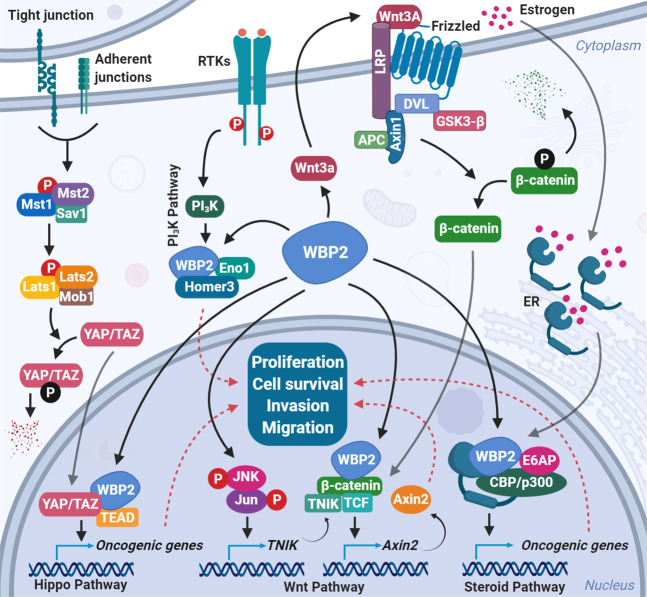
WBP2’s modes of action in cancer. Wnt pathway: WBP2 transcriptionally regulates the TINK expression gene positively through GPS1 and JNK/c-Jun proteins. WBP2-induced Wnt3A-mediated Wnt signaling pathway activation results in an increased β-catenin expression, which in interaction with WBP2, TNIK, and TCF proteins elevates the expression of Axin2 protein. Hippo pathway: Upon inactivation of the Hippo signaling pathway, YAP, and TAZ oncoproteins enter the nucleus and interact with WBP2 to increase the transcription of downstream oncogenic genes. PI_3_K pathway: WBP2 interacts with ENO1 and Homer3, resulting in modulating the ENO1-PI_3_K/Akt signaling pathway. The overall outcome of WBP2 involvement in various pathways is increased cell growth and proliferation, invasion, and metastasis.

To gain better insights into the molecular etiology of cancer, we review the molecular mechanisms by which WBP2 exerts its oncogenic function. WBP2 acts as a transcription coactivator for trans-activating factors like ER/progesterone receptor (PR) and E6-associated protein (E6AP) [4, 5] to regulate the expression of oncogenic proteins such as YAP, cyclin D1, and c-Yes [11]. Together with the more recent involvements of WBP2 in epidermal growth factor receptor (EGFR), Wnt, Hippo, and phosphatidylinositol 3-kinase (PI_3_K)/Akt signaling pathways, the modes of action of WBP2 are summarized in Fig. 2.

#### Steroid signaling pathway

ER/PR play pivotal roles in the initiation and progression of breast cancer [19]. ER and PR are present in 65–80% of breast cancers. ER^+^/PR^+^ cancers tend to grow less aggressively resulting in the formation of low-grade tumors. Treatment options include the classical selective estrogen receptor modulators such as tamoxifen that binds to ER and blocks the binding of estrogen to ER [20]; the aromatase inhibitors, which reduces the amount of estrogen in the body [21]; and the selective estrogen receptor degrader or downregulator such as fulvestrant that binds to ER and causes its degradation [20]. The prognosis of hormone-sensitive breast cancers is generally good with a 5-year survival of ~90% [22].

Upon ligand binding, ER and PR shuttle into the nucleus and trans-activate the transcription of genes important for breast tumorigenesis [23, 24]. Different proteins have been discovered as ER and PR coactivators, for example SRC3 [25], CBP-p300 [26], CARM1 [27], and E6AP [5]. The steroid signaling pathway promotes cell proliferation, invasion, and migration, contributing to breast cancer initiation and progression [24].

WBP2 was reported to be a transcription coactivator for ER/PR. WBP2 forms a complex with E6AP and ER and enhanced the transcriptional activity of ER/PR in a hormone-dependent manner putatively via its recruitment to the ER/PR response elements [5] (Fig. 2). Phosphorylation confers oncogenic property to WBP2 by driving it into the nuclear and promoting its transcription coactivation function [11]. The importance of nuclear WBP2 to breast cancer progression was demonstrated when our lab showed that the WBP2-phosphomimic mutant (Y192–231E), which translocates more effectively into the nucleus, conferred oncogenic properties to non-cancer mammary epithelial cells and aggression to low-grade cancer cells compared with wild-type WBP2 or its phospho-defective mutant (Y192–231F) in vitro and in xenograft models [11]. For example, MCF-7 cells overexpressing phosphomimic mutant of WBP2 underwent epithelial–mesenchymal transition, were more invasive and formed larger tumors in mice compared with WT WBP2. This phenotype of the WBP2-phoshomimic mutant was associated with a more potent coactivation activity on ER-mediated transcription.

#### Wnt signaling pathway

Wnt signaling pathway is important to tissue homeostasis and embryonic development [28]. Aberrant Wnt signaling activity is prevalent in cancer. Adenomatous polyposis coli (APC) mutation and upregulation of constitutively active β-catenin contribute to colon tumorigenesis [29]. Hyperactivity of Wnt signaling has been shown in other cancers, such as breast cancer [28], particularly in TNBC cases [30]. In the absence of Wnt ligand, β-catenin is phosphorylated and targeted for ubiquitination-mediated degradation in the cytoplasm. β-catenin is stabilized in the presence of ligand and translocated into the nucleus, where it coactivates the T-cell factor (TCF)/lymphoid enhancer factor transcription factors [31].

The first hint of a potential role of WBP2 in Wnt pathway was the observations that WBP2 overexpression stabilized β-catenin and activated TCF reporter activity in breast cancer cells [11] (Fig. 2). This was associated with the upregulation of Wnt pathway target genes, like cyclin D1, c-Myc, Bcl-2, vimentin, EGFR-related proteins like extracellular signal-regulated kinase 1/2, focal adhesion kinase and Akt, and downregulation of p21 and p16 proteins [11]. The role of WBP2 in the regulation of cell cycle checkpoint proteins was supported by a subsequent study by Ren et al. [32] who demonstrated that WBP2 upregulated cyclin D1 and cyclin-dependent kinase 4 and downregulated p21 protein in breast cancer. These effects were concomitant with WBP2-mediated G1/S transition, which presumably drives cell cycle progression [32].

A glimpse into the global mode of action of WBP2 in Wnt signaling was revealed through the work of our group in which RNA-seq was employed to ascertain the pervasiveness of WBP2 in Wnt signaling [33]. More than 80% of the Wnt-induced genes in MDA-MB-231 TNBC cells were demonstrated to be dependent on WBP2. In the same study, mass spectrometry-based proteomic analysis indicated that WBP2 primed the molecular soil of TNBC cells by inducing the expression of genes necessary for the TCF transcriptional activity even in the absence of Wnt ligand. One such gene was Axin2 and the WBP2/Axin2 signaling axis was shown to be necessary for Wnt-induced cell proliferation and growth of TNBC cells (Fig. 2). This was an unexpected finding as Axin2 has always been assumed to be a tumor suppressor like the better-known isoform Axin1 [34].

The mechanism of action of WBP2 in promoting breast cancer was elucidated to be dependent on Wnt-induced nuclear entry and interaction of WBP2 with β-catenin, which complexes with TCF to activate gene transcription [13]. This is similar to the mode of action of WBP2 in ER signaling where WBP2 associates with E6AP/p300 to promote ER transcription [4, 5] and reiterates the nuclear role of WBP2 as an oncogenic transcription coactivator.

However, like β-catenin, a large proportion of WBP2 resides in the cytosol. It is conceivable that WBP2 possesses nonnuclear functions that remain to be discovered. This notion is supported by the results arising from the interrogation of the proteomics data against the RNA-seq data which showed that 30% of the WBP2-target proteins were regulated at the mRNA level, whereas the majority were regulated at the posttranscriptional or posttranslational level [33].

#### Hippo signaling pathway

Hippo signaling is a critical signal transduction pathway with an evolutionarily conserved role in regulating organ size and organogenesis mainly through cell contact inhibition process. In addition, it functions as a tumor suppressor by suppressing YAP and TAZ oncoproteins [35–37]. In the presence of stimuli, e.g., cell/cell contact, and the subsequent phosphorylation and activation of downstream tumor-suppressive mammalian sterile 20-like 1/2 (Mst1/2), Salvador 1, large tumor suppressor kinase 1/2 (Lats1/2), and Mps one binder kinase activator-like 1A (Mob1) complexes, YAP and TAZ transcription coactivators are sequestered in the cytoplasm, leading to the contact inhibition-mediated suppressed cell growth. Otherwise, YAP and TAZ proteins actively enter the nucleus to enhance the transcription of oncogenes, in cooperation with transcriptional enhanced associate domain (TEAD) transcription factor, which eventually drives cell growth and proliferation [38].

Chan et al. demonstrated that TAZ protein interacts with the carboxyl terminus (C-terminus) PY motif of WBP2 in a WW-dependent manner, and that the binding with WBP2 was necessary for the oncogenic potential of TAZ [12]. In addition, WBP2 cooperated with YAP ortholog Yorkie in *Drosophila* *melanogaster* (*D. melanogaster*) and increased Yorkie’s transcriptional coactivator activity and thereby the growth of the *D. melanogaster* wing [39]. Moreover, a genome-wide screen by Walko et al. revealed that WBP2 acts via TEAD transcription factors to drive the clonal expansion of normal and malignant human epidermal stem cells [16].

Despite the notable role of WBP2 in the Hippo signaling pathway, it is not known whether WBP2 is regulated by the core kinase cassette (Mst1/2 and Lats1/2) of the Hippo pathway. This is being investigated in our lab.

#### PI_3_K/Akt signaling pathway

PI_3_K/Akt is a well-studied intracellular signaling pathway responsible for some hallmarks of cancer including cell survival and metabolism. Dysregulated PI_3_K/Akt signal transduction is not uncommon in human cancers such as breast [40], lung [41], and glioma [42, 43] and is a cause of drug resistance [44]. Numerous clinical trials on PI_3_K-targeted therapeutics are ongoing [45], highlighting the clinical significance of PI_3_K/Akt pathway in targeted therapeutics.

WBP2 recently emerged as a molecular player in PI_3_K/Akt-mediated oncogenic properties. Consistent with our observation that WBP2 induced the activation of Akt protein in breast cancer [11], Chen et al. reported that WBP2 interacted with α-enolase (ENO1) and homer protein homolog 3 (Homer3) and that the ENO1-PI_3_K/Akt signaling axis drove the proliferation and migration of glioma cells [17].

Taken together, WBP2 exerts its oncogenic function by activating a variety of signaling pathways, driving tumorigenesis and cancer progression/migration in a wide range of human cancers. The ability of WBP2 in activating a myriad of oncogenic pathways indicates that WBP2 is a putative novel biomarker and drug target for WBP2^+^ cancer.

## Multilayer regulation of the WBP2 oncogene

Understanding how the pleiotropic WBP2 oncogene is regulated has a significant impact on the management of cancer. Like most important genes, WBP2 is tightly regulated via a complex variety of mechanisms at the transcription, posttranscription and posttranslation levels [46, 47] to provide a sophisticated but necessary control for fine-tuning the expression and activity of critical genes in the cell.

### Transcriptional regulation of WBP2 by an oncogenic transcription factor

Analysis of the gene copy number alterations and mRNA expression of the WBP2 gene in multiple large-scale breast cancer datasets such as The Cancer Genome Atlas (TCGA) [48] and METABRIC [49, 50] indicated that WBP2 is frequently amplified (4.1–25%) or gained (0–31.7%) in breast cancer patients, whereas deletion was barely present [33]. Other cancer types that showed upregulation of WBP2 gene expression in the TCGA database include kidney renal papillary cell carcinoma, kidney renal clear cell carcinoma, and thyroid carcinoma.

**Fig. 3:**
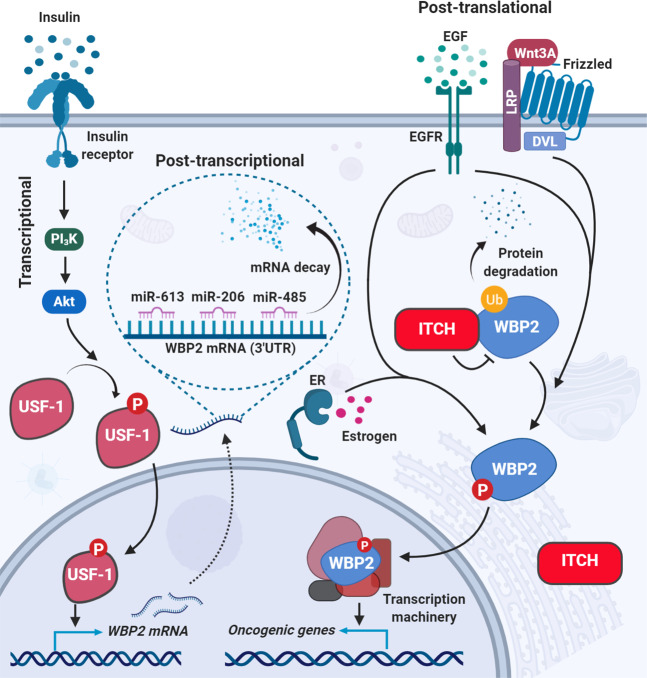
WBP2 regulation in breast cancer. Various modes of WBP2 regulation in breast cancer are Transcriptional: WBP2 is regulated at the transcriptional level through insulin-induced USF-1 transcription factor phosphorylation. Phosphorylated and activated USF-1 enhances the transcription of the WBP2 gene. Post-transcriptional: WBP2 is intensively regulated at the posttranscriptional level through direct binding of miR-613, miR-485, and miR-206 to the WBP2 3′-UTR. Post-translational: Cross talk between Wnt and EGFR signaling pathways phosphorylates and protects WBP2 from ITCH E3-ubiquitin ligase-mediated degradation. This phosphorylation is important for the WBP2/β-catenin cooperation to drive TCF-mediated transcription, as previously shown in Fig. 2. The EGFR/ER signaling pathways cross talk also leads to WBP2 phosphorylation, thereby promoting its translocation to the nucleus to transcriptionally drive the expression of ER/PR-responsive genes.

Since WBP2 protein is well established to be overexpressed in breast cancer [13], we reasoned the transcription factors that drive WBP2 expression would have contributed to this phenotype, at least in part. Testing this hypothesis led to the identification of upstream factor-1 (USF-1) as a novel, oncogenic transcription factor for WBP2 [51]. ChIP experiments showed that USF-1 drives WBP2 transcription by binding to the E-box of WBP2 promoter, resulting in an increase of WBP2 transcript, protein level, and breast cancer cell proliferation [51] (Fig. 3). Survival analysis revealed USF-1 to be a prognostic factor-higher expression of USF-1 correlated with poorer disease-free and overall survival in patients.

Our lab further showed that USF-1 phosphorylation/activation is necessary for its transcriptional activation of WBP2 expression and the former was mediated by the PI_3_K/Akt pathway in response to insulin stimulation [51]. Taken together with the previous findings that WBP2 positively regulates PI_3_K/Akt pathway, the presence of a positive feedback loop between PI_3_K/Akt and WBP2 is envisaged to drive cancer processes.

### Posttranscriptional regulation of WBP2 by miRNAs

Following transcription, RNAs are further regulated by a number of molecules, particularly the different RNA-binding proteins [52] and non-coding small RNAs [53] to provide additional layers of control especially for genes that are potent phenotypic determinants. Through publicly available in silico tools, WBP2 was predicted to be targeted by miRNAs. Evidence showed that the miR-613, miR-206, and miR-485 directly target the WBP2 transcript at its 3′UTR region. The above studies further showed that miRNA-mediated downregulation of WBP2 resulted in cell cycle arrest, apoptosis, and decreased colony formation rate and proliferation [14, 18, 32] (Fig. 3). Extrapolating from the above data, it is conceivable that the upstream inducers of miRNA biogenesis/maturation such as the Hippo signaling components [54] are negative regulators of WBP2.

Clinically, the importance of these WBP2-targeting miRNAs is supported by meta-analysis which revealed a considerable downregulation of miR-206 and miR-485 in breast cancer with a corresponding negative correlation with WBP2 expression (Supplementary Fig. 1A–D). Notwithstanding the moderate but statistically significant correlation coefficients, the clinical data supports the observed regulation of WBP2 posttranscriptionally by miRNAs in the lab. However, the authors caution that correlation analyses at the transcript level may not be accurate due to the concept that this review paper reiterates, namely the mRNA level of WBP2 does not necessarily reflect its protein expression due to the extensive posttranscriptional/translation modifications WBP2 undergo. A proper study correlating the level of candidate miRNAs to WBP2 protein level e.g., via IHC would be more meaningful.

### Posttranslational regulation of WBP2

Posttranslational modification of proteins is another major layer of control through which cells fine-tune the biochemical pathways to achieve the desired cellular outcomes. Protein posttranslational modifications are probably the most diverse, involving a myriad of molecular switches such as phosphorylation, acetylation, and ubiquitination that control protein expression, conformation, activity, localization, binding, and/or stability [55–57].

#### WBP2 protein phosphorylation

Tyrosine phosphorylation was the first posttranslational modification of WBP2 to be discovered in a phosphoproteomics study of breast cancer development [10]. It is a key regulatory switch for the WBP2 function. For example, E2-induced tyrosine phosphorylation of WBP2 stimulated its nuclear localization and promoted ER activity [11], while Wnt-induced tyrosine phosphorylation of WBP2 disrupted ITCH/WBP2 binding, stabilizing WBP2 that subsequently cooperated with β-catenin to drive TCF-mediated transcription [13]. Direct activation of EGFR by EGF, cross talk with EGFR by estrogen and Wnt ligands were responsible for tyrosine phosphorylation of WBP2. This indicates that EGFR/WBP2 is a central node for signaling inputs and functions.

#### Ubiquitination and proteasomal degradation

Ubiquitination-mediated proteasomal degradation is a key regulatory mechanism used widely for many signaling systems. Ubiquitination process is composed of three enzymatic steps carried out by E1 ubiquitin-activating, E2 ubiquitin-conjugating, and E3 ubiquitin ligase enzymes. The ubiquitinated protein is eventually marked to be degraded by the proteasomal degradation [58]. For example, β-catenin is phosphorylated in a destruction complex composed of APC, Axin1, casein kinase 1 α, and glycogen synthase kinase 3β, thereby ubiquitinated by beta-transducin repeat-containing protein (β-TRCP) [59]. Interestingly, Lats1/2-Mob1-induced phosphorylated YAP and TAZ proteins are also substrates of β-TRCP E3 ubiquitin ligase [60]. Could WBP2, which is intricately associated with Wnt and Hippo signaling, be a target of proteasomal degradation?

Following the establishment of WBP2 as an oncogene, we attempted to identify the binding partners of WBP2 to elucidate how WBP2 acts and/or is regulated. WW domain-containing E3-ligase ITCH was discovered, via 2 different approaches (mass spectrometry and yeast-two-hybrid), to bind to and degrade WBP2 through an ubiquitin/proteasomal pathway (Fig. 3). WBP2 expression was profoundly increased when normal mammary epithelial and cancer cell lines were subjected to ITCH knockdown or treatment with proteasomal inhibitors [13].

Following the discovery that ITCH downregulates oncogenic WBP2 expression, we reasoned that a loss-of-function (LOF) mutation in ITCH would abolish its effect on WBP2 expression. Indeed, an artificial ligase-dead ITCH C830A mutant failed to abolish WBP2 expression, molecular, and cellular function. This led us to pursue further to look for clinical ITCH mutations in the COSMIC database and examine their functional phenotypes. Nine potential LOF mutations were found, of which six were in the E3-ligase domain. All nine mutants were created and systematically characterized for LOF in downregulating WBP2 expression [13]. Three positive hits were obtained—E184K, R833C, and E855K. The latter two reside in the E3-ligase domain and occurred at a frequency of 3.1% and 1.7% in TNBC, respectively. All three mutants failed to degrade WBP2, with E855K showing the strongest phenotype. Follow-up studies revealed that E855K ITCH mutant indeed could not abolish WBP2-mediated cancer growth in vitro and in vivo [13].

Meta-analysis of the TGCA database revealed that the expression of ITCH does not change significantly in cancer (Supplementary Fig. 1E). Despite the relatively low incidence, it highlights the importance of ITCH LOF mutations in cancer biology. Collectively, the evidence further supports the notion of WBP2 as an oncogene that is downregulated by ITCH tumor suppressor-mediated proteasomal degradation in at least a subset of aggressive breast cancers. Future clinical studies correlating the ITCH mutational status with WBP2 protein expression would offer greater clarity on the prevalence of the ITCH/WBP2 signaling axis on breast cancer.

In the same study [13], other WW domain-containing E3 ubiquitin-protein ligase 1 (WWP1), WWP2, Ubiquitin A-52 residue ribosomal protein fusion product 1 (UBA52), E3 ubiquitin-protein ligase NEDD4-like (NEDD4L), and WWE domain-containing 1 (HUWE1) E3 ligases were also identified as potential binders of WBP2. Although they remained to be validated, the data suggest that proteasomal degradation is a key posttranscriptional rheostat for WBP2 expression.

#### WBP2 molecular interactions and rational drug design

**Fig. 4:**
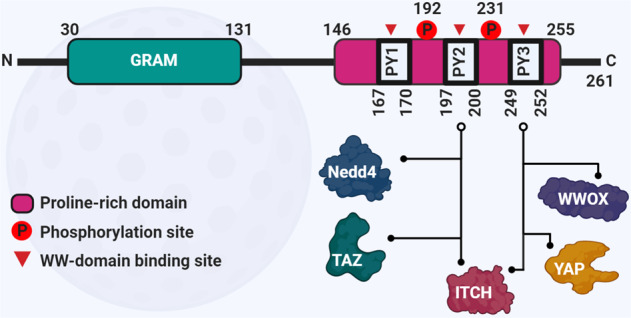
Structure of WBP2 protein. The N-terminal GRAM domain is followed by three PPxY (PY) motifs in the C-terminal region. PPxY is described as P = Proline, x = any amino acid, and Y = Tyrosine. These PY motifs recognize and interact with the WW domain(s) of target proteins, including YAP, Taz Nedd4, and WWOX. Tyr 192 and Tyr 231 are the phosphorylation sites, which are important in regulating the WBP2 activity. The GRAM domain might be important for the integrity and full function of the protein [48].

Mapping protein interaction sites and elucidating how these sites are regulated have proven to be a viable approach to the development of biologics such as therapeutic peptides, aptamers as well as small molecules that disrupt protein binding, molecular, and hence cellular functions [61–63]. Biochemical evidence highlights the importance of the C-terminal of WBP2 to its interactions with WW domain-containing proteins (Fig. 4). The WW domains of proteins interact with one or more PY motifs located at the C-terminus of WBP2 to control molecular and cellular processes [3]. For example, TAZ and Nedd4 WW domains interact with the WBP2 PY2 motif [12, 64], ITCH WW domains 1 and 3 interact with WBP2 PY2 and PY3 motifs to regulate cancer growth [13], while WWOX and YAP WW domains interact with PY3 [12, 65] (Fig. 4). WBP2 also interacts with proteins that lack WW domain (Fig. 1). This implies that the WBP2 protein interaction network is not restricted to WW-containing domain proteins and may include proteins with other domains that bind proline-rich sequences such as EVH1 domain of vasodilator-stimulated phosphoprotein (VASP) or SH3 domain of Abl [66].

The PY motifs of WBP2 are in close proximity to the tyrosine phosphorylation sites (Fig. 4). Moreover, wild-type WBP2 was less susceptible to ITCH-mediated degradation in the presence of WNT3A compared with WBP2 phospho-defective mutant, while WBP2-phosphomimic mutant displayed increased stability and reduced polyubiquitination in the presence of ITCH compared with wild-type WBP2 [13]. In both cases, the increased stability of WBP2 could be attributed to the diminished interaction between WBP2 and ITCH that appears to be negatively regulated by WBP2 phosphorylation. While isothermal calorimetry (ITC) confirmed that ITCH binds WBP2 directly, no significant differences in the dissociation constant (Kd) between the phosphorylated and non-phosphorylated peptides of WBP2 with ITCH WW domain was observed [13]. Although this implies that phosphorylation does not affect binding between the WW domains 1 and 3 of ITCH and PY2 and PY3 motifs of WBP2, it does not rule out the potential effect of phosphorylation on the conformation of WBP2 that might influence such interactions.

Is there a role for PY1 motif in mediating WBP2 binding to its partners? The PY1 motif (PPGY) of WBP2 has a different core sequence compared with its neighboring PY2 and PY3 motifs (PPPY). It is an interesting notion that proteins containing other proline-rich binding domains such as EVH1 and SH3, which have a different preference for motif sequence from WW domain, may be identified as WBP2 binding partners in the future, thus providing further insights into the molecular etiology of cancer. To date, an EVH1-containing protein Homer3 has been shown to interact with WBP2, which drives the PI_3_K pathway-mediated oncogenic properties of WBP2 in cancer [67].

WBP2 belongs to a family of transcription coactivators which play roles in different oncogenic signaling pathways and have attracted much attention as potential drug targets. For instance, steroid receptor coactivators 1–3 (SRC1–3) proteins are important oncogenic coactivators in hormone-positive breast cancer. Recently, SI-2 small molecule has been reported to selectively reduce the SRC3 protein concentration and its corresponding transcriptional activities. This leads to the significant tumor growth inhibition in breast cancer cell lines and xenograft mouse model [68].

Can WBP2 be exploited as a drug target? Its transcription coactivator role in the nucleus argues against WBP2 as a viable drug target. Like SRC however, WBP2 is predominantly in cytosol and a small population of it translocate to the nucleus only when triggered by oncogenic signals. This makes targeting WBP2 via cell-permeable agents feasible. Based on the knowledge about WBP2, a few therapeutic approaches are conceivable. One approach is to block WBP2 protein–protein interactions. As an illustration, WBP2 sequence-derived peptides comprising the PY motifs and/or phosphorylation sites could be designed as competitive inhibitors against the functional C-terminus of WBP2 [69]. A key challenge in this approach is the effective delivery of the peptide into the cells, which could be circumvented by tagging a cell-permeable sequence to the therapeutic peptide [70]. Preliminary studies in our lab support the notion that this approach can inhibit certain molecular functions of WBP2 (data not shown). Alternatively, small molecules that disrupt molecular interactions between WBP2 and binding partners could be developed.

**Fig. 5:**
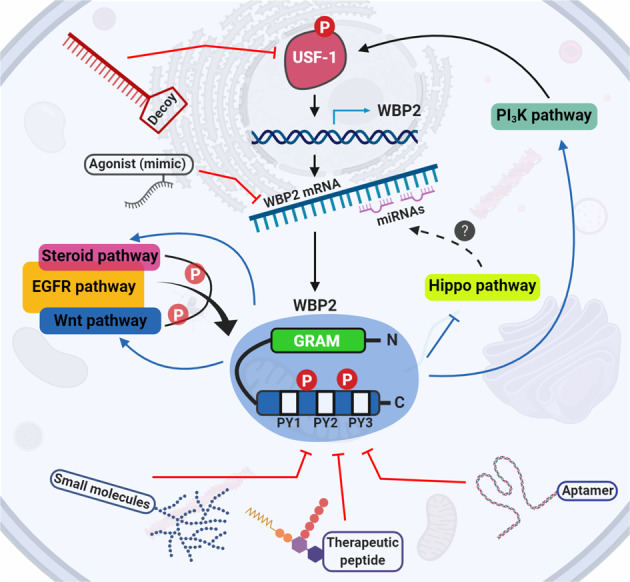
WBP2 signaling network and rational drug design. The ability of WBP2 in activating a myriad of oncogenic functions through PI_3_K/Akt, EGFR, ER, and Hippo proposes a multilayer targeting approach in WBP2-positive cancer cases for downregulating WBP2 and thereby its oncogenic properties. Decoy oligonucleotides against the USF-1 oncogenic transcription factor could block WBP2 expression at the transcriptional level. miRNA mimics could further repress the expression of WBP2 by targeting its 3′UTR. WBP2’s functions could be controlled at the protein level via designing therapeutic biologics such as aptamers and therapeutic peptides as well as small molecules against WBP2’s C-terminal region.

WBP2 may also be targeted indirectly at the transcriptional level using decoy oligonucleotides [71] to trap the USF-1 transcription factor, while WBP2 mRNA could be targeted using miRNA mimics [72] (Fig. 5). Another avenue is to activate ITCH E3 ubiquitin ligase that should lead to downregulation of WBP2 expression and cancer growth. This can be achieved via protein-targeting chimeric molecules (PROTACs), which are composed of a ligase-recruiting ligand and a short linker to a second ligand that binds the target protein [73]. PROTAC-1 has been shown to successfully degrade methionine aminopeptidase 2 oncoprotein by recruiting Skp, Cullin, F-box containing complex (SCF) E3 ubiquitin complex [74]. While this method avails a new avenue to exploit E3 ligase for cancer therapy, it remains to be proven in vivo.

The key function of transcription factors and coactivators is the assembly of transcriptional complexes that not only execute transcription but also epigenetic regulation such as chromatin remodeling to promote transcription. Supporter of activation of yellow protein is one of the most well-known coactivators with ability to assemble chromatin-remodeling factor Brahma and the transcription initiation factor TFIID [75]. WBP2 has been shown to recruit histone acetyl transferase (HAT) p300, an epigenetic regulator, to ER response elements, and presumably enhances transcription of target genes by promoting chromatin relaxation through histone acetylation [4]. Histone deacetylase (HDAC) on the other hand represses the action of HAT and suppresses transcription. Impairing the function of HDACs dysregulates the expression of genes involved in cancer initiation and progression and is an actively researched area for cancer therapy [76]. For example, a phase III trial showed that breast cancer patients benefitted from treatment with a HDAC inhibitor tucidinostat [77, 78]. However, HDAC inhibitors have selectivity for HATs with distinct histone acetylation marks e.g., H3K9 and H4K12; and therefore, dysregulation of different sets of genes [48, 79]. It remains to be investigated what exact histone modification/s WBP2 causes, if any. More high-resolution research on WBP2 and chromatin remodeling will be needed in order to exploit HDAC inhibitors for targeted therapeutics of WBP2-positive breast cancer.

Notwithstanding the above approaches remain to be tested even in the lab, and while it will take many more years to move into preclinical and clinical studies, and perhaps not, we expect the drug development efforts against WBP2 transcription coactivator to emerge within the next 5 years.

## Translational and clinical significance of WBP2

Cancer fatalities is a major public healthcare problem; key pillars to manage this disease, besides prevention, are early detection and precision medicine [80].

Targeted therapeutics, has advanced the management of cancer. A classic example is the FDA approval of the use of humanized monoclonal antibody trastuzumab/Herceptin for the treatment of HER2^+^ breast cancer in 1998 [81]. This is followed by many other drugs, such as Gleevec [82] and Gefitinib [83] that target Bcr-Abl and EGFR for chronic myelogenous leukemia and lung cancer, respectively. About four decades since the 1980s, targeted therapeutics has reached its full blossom. Today, there are more than 800 therapeutic molecules in late-stage oncology pipeline, up 77% when compared with 2008 (https://www.iqvia.com/insights/the-iqvia-institute/reports/global-oncology-trends-2019).

A key challenge in cancer therapeutics is intrinsic/de novo and acquired drug resistance. Identifying the mechanisms of drug resistance is an important endeavor. The work by Chen et al. implicates WBP2 in the resistance of MCF-7 cells to doxorubicin. Overexpression of WBP2 promoted its interaction with ER, which in turn enhanced ER-mediated transcription of multidrug resistance gene (MDR1) [84].

With the development of targeted therapeutics comes the advent of companion diagnostics for precision medicine. Stratification of patients using biomarkers that predict which patients are more likely to respond to a specific drug improves patient outcomes and healthcare costs. Trastuzumab in combination with chemotherapy has helped manage HER2^+^ patients. However, only 30–50% of the patients respond to this treatment regimen [85, 86]. This highlights the urgent need for better or complementary predictors of therapeutic response.

WBP2 is not only a downstream substrate of EGFR/HER2 signaling. It is also a potential predictive biomarker of response to trastuzumab-based neoadjuvant therapy in HER2^+^ breast cancer patients [15]. Exogenous WBP2 enhanced the inhibitory effect of trastuzumab on cell proliferation and cell cycle in vitro and in xenograft models. A multicenter retrospective study revealed that breast cancer patients that were HER2^+^ and had high WBP2 expression responded significantly better to trastuzumab-based neoadjuvant therapy. This is most profound in patients <50 years old, who recorded a pathologic complete response of about 80% compared with 44.8% in HER2^+^ patients with no further stratifications [15].

## Concluding remarks

WBP2 is proving to be an emerging and potent oncogene. Cancer is the predominant disease area in WBP2 research and it looks set to grow over the next 10 years. At the molecular level, WBP2 exerts its oncogenic effect by activating multiple signaling pathways including the steroid, EGFR, Wnt, PI_3_K/Akt, and Hippo signaling pathways. At the cellular and physiological levels, aberrantly high expression of WBP2 results in cell proliferation, anchorage-independent growth, invasion and migration, and tumorigenesis. WBP2 is regulated in a sophisticated and complex manner involving multiple levels of control. Understanding how WBP2 is regulated can be exploited for rational drug design.

### WBP2 protein level as a more accurate measurement of expression for clinical use

**Fig. 6:**
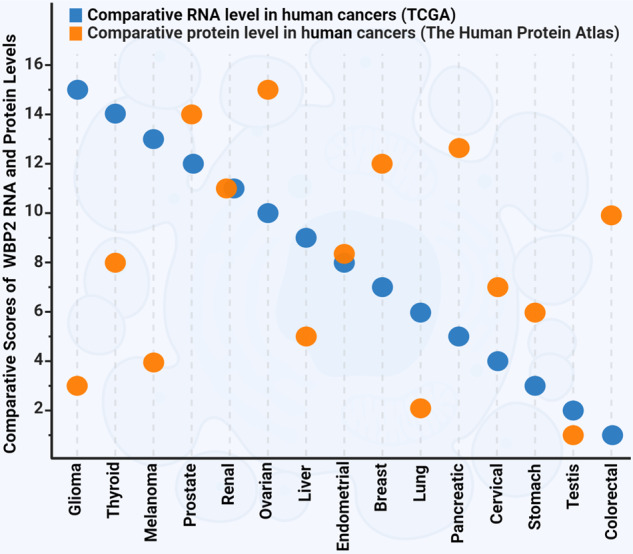
Comparative overview of the expression levels of WBP2 mRNA versus protein in human cancers. The RNA expression was obtained from RNA-seq data of The Cancer Genome Atlas (TCGA) [95]. The 15 human cancers were sorted and scored in terms of the WBP2 RNA level from 15 (the highest expression) to 1 (the lowest expression). The protein expression was obtained from The Human Protein Atlas database [96]. Similarly, the sorting and scoring method was carried out for human cancers in terms of WBP2 protein level. Scatter plot was used to show the RNA and protein scores for each cancer. Cancers with high level of WBP2 mRNA such as glioma, melanoma, and thyroid showed low protein expression level, while pancreatic and colorectal cancers with high level of WBP2 protein display low mRNA expression. The unit for the RNA-seq data is reads per kilobase of transcript per million mapped reads (FPKM). This unit represents the relative expression of the WBP2 transcript proportional to the number of cDNA fragments that originate from it. The protein expression data is based on the percentage of the patients who have medium or high expression of WBP2 in each cancer.

Although WBP2 aberrations occur at the genomic and transcript levels, evidence emphasized that WBP2 expression should be evaluated at the protein level for it to be clinically useful. This is because the expression of WBP2 protein and mRNA were observed to be largely disproportional. Quantitative PCR of a panel of 17 cell lines revealed at best 50% concordance between WBP2 transcript and protein levels [13]. As a confirmation, we sorted 15 human cancers in terms of the WBP2 RNA expression level based on TCGA database—glioma showed the highest RNA level (scored of 15) and colorectal cancer the lowest (scored of 1). The same was performed based on WBP2 protein expression level using The Human Protein Atlas database [80]. Cancers with high WBP2 mRNA levels such as glioma, melanoma, and thyroid had low protein expression level, whereas pancreatic and colorectal cancers with a high WBP2 protein level display low mRNA expression (Fig. 6). Interesting, the other three cancers (breast, ovarian, and prostate) with high WBP2 protein level are hormonally regulated.

Moreover, the level of WBP2 protein in normal mammary epithelial and breast cancer cell lines could be elevated with the treatment of proteasomal inhibitors or silencing of ITCH E3 ligase [13]. Collectively, these evidences highlight that posttranslational modification is a predominant determinant of WBP2 expression. Genomic analysis/measurement of WBP2 is of limited use in the clinical setting as it may misguide diagnosis and prognosis.

It is further recommended that expression of WBP2 be assessed by IHC, immunofluorescence, or immunocytochemistry techniques as the subcellular localization of WBP2, e.g., nuclear WBP2, can be a diagnostic feature since the oncogenic phosphomimetic WBP2 mutant resides in the nucleus and nuclear staining of WBP2 has been largely observed in cancer but not normal tissues [13].

### An expanding network of targetable signaling pathways associated with WBP2 oncogenic function

**Fig. 7:**
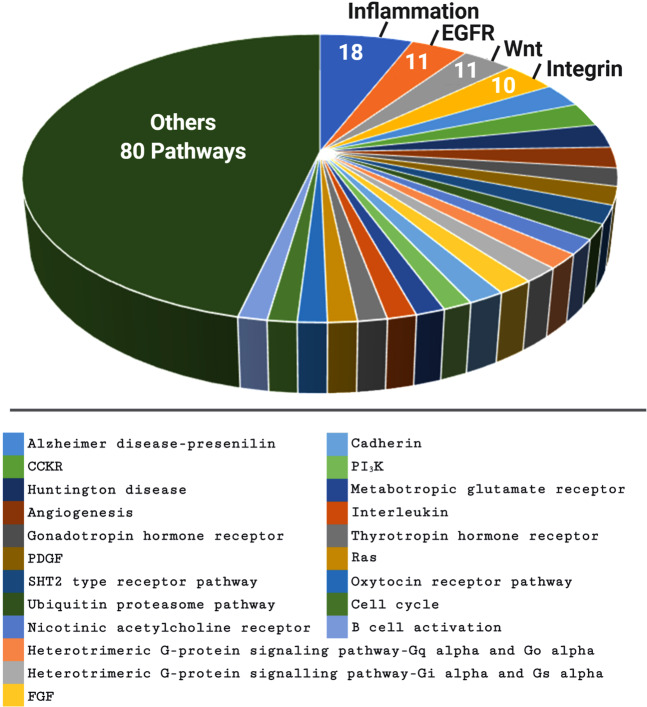
The potential WBP2 signaling pathway network. In silico analysis of the gene copy number variation (CNV) of normal versus breast cancer tissues in The Cancer Genome Atlas (TCGA) [95] database reveals that the upregulation of WBP2 was associated with the amplification of chromosome 17q. Enrichment analysis of the list of genes in the amplified region of chromosome 17q using the PANTHER gene ontology database uncovered a network of key signaling pathways associated with WBP2.

WBP2 is involved in an increasing number of signaling pathways, human cancers, and even canine oral cancer [87]. The robust oncogenic effect of WBP2 as a result of its transcription coactivator role suggests that WBP2 has a wider role in other oncogenic signaling cascades, human cancer types and diseases. In silico gene copy number variation (CNV) analysis performed in our lab reveals that the upregulation of WBP2 was associated with chromosome 17q (C17q) amplification. Enrichment analysis of the list of genes in the amplified region of C17q using the PANTHER gene ontology database [88] indicates that WBP2 participates in Wnt, EGFR, NF-κB/inflammation, and integrin signaling pathways (Fig. 7), of which WBP2 protein has already been implicated in the former two [11, 13, 33]. NF-κB pathway is a well-characterized transduction system tightly associated with cancer hallmarks [89]. Similarly, the integrin signaling pathway is a key molecular cascade involved in cancer cell survival and chemoresistance [90].

This raises a testable hypothesis that WBP2 plays a role in the NF-κB and integrin signaling pathways to regulate tumor microenvironment and remodeling of the extracellular matrix to facilitate tumorigenesis, angiogenesis, and metastasis. This has implications in targeted therapeutics.

### WBP2 in human diseases other than cancer

Does WBP2 play a role in diseases beyond human cancer? The WBP2-associated hearing and fertility disorders reported support this notion. Our in silico analysis (Fig. 7) implicated WBP2 in neurological disorders like Huntington’s and Alzheimer’s disease. On the other hand, mass spectrometry revealed that WBP2 interacts with coatomer protein complex subunit alpha/beta1/beta2/epsilon/gamma, WWP2, DEAD-box helicase 17 [13], VASP, glial fibrillary acidic protein and vimentin [17]—proteins that play roles in immune and hematological disorders. Finally, USF-1-mediated regulation of WBP2 in response to insulin stimulation raises the possibility of WBP2’s involvement in insulin signaling-related diseases such as insulin resistance and diabetes [91].

The conditional WBP2 knockout mouse line generated by the International Mouse Phenotyping Consortium showed several phenotypes [92, 93]. Four are related to the nervous system, two to the adipose tissue/metabolic system, and one to the hematopoietic system. These phenotypes were broadly consistent with the human diseases insinuated by published data and our in silico analysis. While these are hypothetical scenarios, they present exciting avenues for investigating the role of WBP2 in a wider scope of human diseases.

### Two is better than one: WBP2 as co-companion diagnostics with HER2 for trastuzumab-based cancer therapy

The “one biomarker and one drug” paradigm in precision medicine is proving to be lacking. For example, the use of HER2-based diagnostics to guide trastuzumab treatment falls short of producing a good response rate. We see a trend towards multiplex companion diagnostics for cancer therapeutics. The combined use of HER2 and WBP2 as companion diagnostics for trastuzumab is one such case. Due to the roles WBP2 play in other signaling systems, it may not be surprising that WBP2 also regulates cellular response to other targeted therapeutics.

Moving forward into the next decade, we expect to see WBP2 playing a wider role in human cancers and diseases, signal transduction pathways, and precision medicine. Drug development efforts against WBP2 should emerge and contribute to the pipeline of biologics and small molecules against cancer and other metabolic diseases.

## Supplementary information

Supplementary Figure 1

Supplementary Figure 1-Legend
