# Supplementary figures and images for: The emerging roles of WBP2 oncogene in human cancers
Source: Oncogene. 2020 May 11;39(24):4621–35. doi: 10.1038/s41388-020-1318-0 (PMC7286818; doi:10.1038/s41388-020-1318-0)

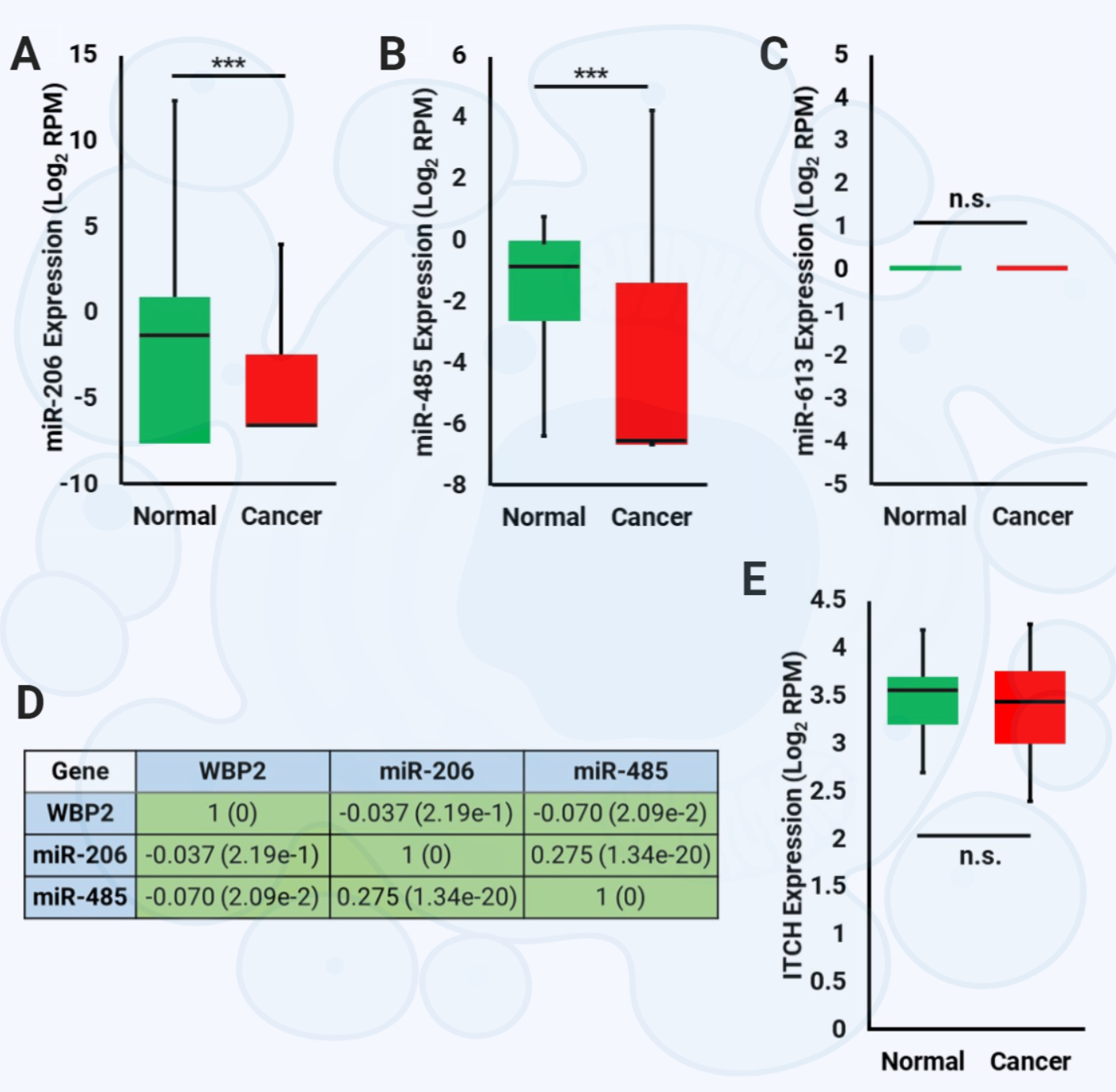

Supplement: Supplementary file 1 — Supplementary Figure 1 [file 41388_2020_1318_MOESM1_ESM.jpg]
