# Supplementary material for: The emerging roles of WBP2 oncogene in human cancers
Source: Oncogene. 2020 May 11;39(24):4621–35. doi: 10.1038/s41388-020-1318-0 (PMC7286818; doi:10.1038/s41388-020-1318-0)
Supplement: Supplementary file 2 — Supplementary Figure 1-Legend [file 41388_2020_1318_MOESM2_ESM.docx]

**Supplementary Figure 1.** Analysis of TCGA RNA-seq data using ENCROI database, showing the down-regulation of **(A)** miR-206 and **(B)** miR-485, and no significant changes in **(C)** miR-613 in 1085 breast invasive carcinoma and 104 normal samples. **(D)** A correlational analysis among the transcript levels of WBP2 and its targeting miRNAs, miR-206 and miR-485 in 1085 breast invasive cancer samples. **(E)** Analysis of TCGA RNA-seq data using ENCROI database, showing no significant changes in ITCH transcript level. The data obtained from ENCORI database based on TCGA data analyses (<http://starbase.sysu.edu.cn/>).
